# Evaluation of contrast sensitivity in visually impaired individuals using K-CS test. A novel smartphone-based contrast sensitivity test–Design and validation

**DOI:** 10.1371/journal.pone.0288512

**Published:** 2024-02-08

**Authors:** Vasileios Karampatakis, Eleni P. Papadopoulou, Stavroula Almpanidou, Leonidas Karamitopoulos, Diamantis Almaliotis

**Affiliations:** Laboratory of Experimental Ophthalmology, School of Medicine, Aristotle University of Thessaloniki, Thessaloniki, Greece; LV Prasad Eye Institute, INDIA

## Abstract

**Background:**

To describe the development and investigate the accuracy of a novel smartphone-based Contrast Sensitivity (CS) application, the K-CS test.

**Methods:**

A total of 67 visually impaired and 50 normal participants were examined monocularly using the novel digital K-CS test and the Pelli-Robson (PR) chart. The K-CS test examines letter contrast sensitivity in logarithmic units, using eight levels of contrast from logCS = ~0,1 to logCS = ~2,1 at two spatial frequencies of 1.5 and 3 cycles per degree (cpd). The K-CS test was compared to the gold standard, PR test and intra-session test repeatability was also examined.

**Results:**

The K-CS test in normally sighted was found to agree well with the PR, providing comparable mean scores in logCS (±SD) (K-CS = 1.908 ± 0.06 versus PR = 1.93 ± 0.05) at 1.5 cpd and mean (± SD) logCS at 3 cpd (K-CS = 1.83 ± 0.13 versus PR = 1.86 ± 0.07). The mean best corrected visual acuity of visually impaired participants was 0.67 LogMAR (SD = 0.21) and the K-CS was also found to agree well with the Pelli-Robson in this group, with an equivalent mean (±SD) logCS at 1.5 cpd: (K-CS = 1.19 ± 0.27, PR = 1.15 ± 0.31), 3 cpd: K-CS = 1.01 ± 0.33, PR = 0.94 ± 0.34. Regarding the intra-session test repeatability, both the K-CS test and the PR test showed good repeatability in terms of the 95% limits of agreement (LoA): K-CS = ±0.112 at 1.5 cpd and ±0.133 at 3 cpd, PR = ±0.143 at 1.5 cpd and ±0.183 in 3 cpd in visually impaired individuals.

**Conclusion:**

The K-CS test provides a quick assessment of the CS both in normally sighted and visually impaired individuals. The K-CS could serve as an alternative tool to assess contrast sensitivity function using a smartphone and provides results that agree well with the commonly used PR test.

## Introduction

Visual acuity (VA) testing is the most widely used clinical examination of visual function. VA is most commonly measured using letters of high contrast presented on charts and is widely used for screening, determining a refractive error, and monitoring the progression or the treatment of a disease [1]. However, recent studies show that contrast sensitivity function seems to be more sensitive revealing early signs of vision impairment for a significant number of ocular diseases [2, 3]. Contrast sensitivity (CS) is the ability to detect subtle differences between the luminance of an object compared to its background [4], and discriminating figures without distinct outlines [5]

Vision tests, performed with reduced contrast targets, have been proven to capture more comprehensively the severity of vision loss [6, 7]. Recent data support that small changes in the quality of the retinal image in healthy eyes is best reflected by a corresponding change in low-contrast (LC) than a change in high-contrast (HC) VA [8]. The multiple levels of contrast that are featured in a CS test represent variations very similar to daily visual experiences [9, 10]. Importantly, the quality of vision is highly affected by poor contrast sensitivity function, which can even occur in patients with normal VA [11, 12].

Acquired and inherited ocular diseases are associated with contrast sensitivity abnormalities [13]. The wide spectrum of such diseases includes inherited retinal degenerations, early and advanced diabetic retinopathy, maculopathies or glaucoma [14–17]. Importantly, contrast sensitivity is a sensitive indicator of impaired macular function. In the early stages of age-related macular degeneration (AMD) for instance, contrast sensitivity function in some cases is affected earlier than high contrast VA [18]. CS function loss implies also the risk of a subsequent VA loss in people with geographic atrophy [19]. Furthermore, individuals with inherited retinal diseases (IRDs) often report poor visual function even when VA is relatively stable [20]. In the same line, patients with Stargardt’s disease could also experience significant CS function loss [21]. CS testing might serve as a screening tool for detecting early changes in patients with diabetes mellitus, even before the onset of diabetic retinopathy [22, 23]. The loss of CS function is far more obvious in the presence of central macular edema (CME) in diabetic retinopathy [24]. Even though patients with CME have lower mean letter CS scores compared to patients without CME, this difference may not be clinically significant, depending on the pathology of the photoreceptors and the severity of the edema. Most ocular diseases have a progressive course and the prognosis depends on the time of therapeutic intervention. Hence, clinical tests providing detection of early signs of disease progression are needed to secure a better clinical outcome [25].

Periodic sine-grating (Arden grating test, Cambridge low contrast test, Vector vision’s CSV-1000, etc.) and non-periodic pattern (letters) tests (Pelli-Robson, Mars test, Regan test, etc.) [26] are widely used methods of examing CS by determining patients’ foveal contrast thresholds. The cycles per degree of the gratings and the letters, determine the spatial frequency. The Pelli-Robson contrast sensitivity chart [27] has been proposed as the most commonly used method in clinical practice for assessing CS. The chart is typically viewed from a distance of 1m and combination with other test is recommended [28]. Despite its clinical significance, CS testing has not been widely adopted into clinical practice as traditionally it requires long testing time and specialized laboratory equipment. Hence, the development of a quick and reliable test of the CS function in portable devices such as smartphones may overcome these obstacles.

In the current study, we described the development of a novel smartphone-based CS test and examined its clinical performance against the gold standard PR test in patients with visual impairment and in normally sighted individuals.

## Materials and methods

### Participants

In total, 117 individuals were included in the study;67 were visually impaired and 50 were normally sighted participants. All participants were recruited prospectively from our outpatient unit at the Aristotle University of Thessaloniki, School of Medicine running the LIFE4LV project for patients with visual impairment, which was officially registered at ClinicalTrials.gov. (NCT05184036). Patients were excluded with the following criteria:

- ■ BCVA worse than 1.0 LogMAR,
- ■ High refractive errors, such as hyperopia (+5,00sph), myopia (-5,00sph), or astigmatism (+/- 3,00cyl or higher),
- ■ Previous refractive surgery,
- ■ Significant lenticular changes according to WHO classification
- ■ Cloudy cornea, keratitis, acute uveitis, acute glaucoma
- ■ Patients with cognitive and/or mental impairment
- ■ Pharmacological treatments that could affect their vision, such as epileptic, sedative drugs or the use of mydriatic eye drops the same day before their examination

Approval for this study has been received by the Bioethical Committee, School of Medicine of the Aristotle University of Thessaloniki (code#1.60/21.11.2018). The study was conducted by the rules and regulations of the Declaration of Helsinki. Subjects provided written informed consent before participation. The General Data Protection Regulation GDPR in a research context and the Greek Law of Data Protection were respected.

### Contrast sensitivity examination

#### Pelli-Robson CS test

The Pelli-Robson (PR) CS test is comprised of black Sloan letters arranged in 16 groups of 3 letters and is performed on a white chart measured 59x84cm. The PR chart has a range of contrast levels, from 0.00 log units (100%) to 2.25 log units (0.56%), and each triplet of letters decreases by 0.15 log units. All letters occupy a spatial frequency depending on the testing distance. The chart was also performed at approximately 1.7m, instead of the standard distance of 1m, to achieve a spatial frequency of 1.5 cpd [29, 30]. Given that the spatial frequency of 3 cpd is considered to be the region of peak sensitivity and could provide extra information about the performance of the participants, they were also examined at the appropriate distance to achieve a spatial frequency of 3 cpd [30, 31].

#### Characteristics of the novel K-CS test

The K-CS test is a novel, smartphone-based CS test, designed to examine the contrast thresholds of normal and visually impaired individuals. The test is displayed on the front panel of a smartphone and requires internet access. The application is available for Android devices supporting OS 5.0 or updated versions. The test is based on Weber contrast and examines the letter CS.

The test evaluates the CS function in logarithmic units. Contrast is regulated by the application through the opacity property; by setting the opacity of an element (in our case letters) to the desired value, the required contrast level is achieved. Opacity is defined as the degree to content behind an element is hidden and is the opposite of transparency. Opacity(%) is the contrast ratio multiplied by 100 to get a percentage, where 100% is complete hiding.

The test starts from the easiest contrast threshold and reaches the most difficult for each spatial frequency. To be comparable with the gold standard PR test, the K-CS examines CS function in two letter sizes corresponding to 1.5 and 3.0 cycles per degree, respectively. The K-CS test presents a single row of five, black letters (ex. H, K, N, O, Z, etc.) based on the ETDRS optotype, on a white background screen. Letters are presented horizontally on the device in a randomized order, determined by a computer algorithm. The K-CS test can produce a range of contrast between ~0.1 and 2.06 logarithms of CS. The interstimulus drops in contrast levels consist of steps of ~0.3 log units. LogCS is identified as the lowest contrast threshold for which the individual can discriminate at least 3 out of 5 letters in the aforementioned two spatial frequencies. The results can be saved and transmitted to the attending physician who has been previously authorized by the patient. Hence, the results of each examination may be compared to previously saved results by the physician.

#### Apparatus

A Samsung Galaxy A30S (Android OS 9.0, Mali-G71 MP2; display: Super AMOLED, size: 6.4 inches, 100.5 cm2, resolution: 720 x 1560 pixels– 19.5:9 ratio (~268 ppi density); weight: 169 g) was used for the examination with the K-CS test. The discrete contrast levels (log units) which could be produced by the device were measured using the X-Rite i-ONE precision photometer and are given in S1 **Table.**

#### Examination protocol

All subjects underwent a comprehensive eye examination including measurement of the best corrected visual acuity (BCVA) with the Early Treatment Diabetic Retinopathy Study (ETDRS) chart (Precision Vision, USA, chart 1) under standard clinical conditions. Moreover, other variables such as general health history, including eye disorders that might interfere with CS and also systemic conditions and medication along with the current refractive correction, were recorded as well [32, 33]. Each examinee was examined individually and monocularly. The patients completed the whole examination in a single visit. The Pelli-Robson test was performed by an examiner, while the K-CS test was performed by the participants after the appropriate training, following instructions present on the screen of the smartphone before the examination. The order of testing has been changing systematically and adequate rest time was provided at intervals, if necessary. Both tests, K-CS and PR, were performed in a clinical setting. Subjects were examined with their best refractive correction and head movements were allowed. Only the eye with the BCVA was examined and the other eye was occluded with an eye patch.

The room and the chart illumination were appropriately standardized. There were no windows to minimize the amount of glare. The test was conducted at 100% brightness of the Android smartphone. Within the mobile’s settings, the space-averaged luminance of the screen was approximately 485 cd/m^2^. The background luminance of the PR chart was around 85 cd/m2, which was between the recommended range of 60–120 cd/m2 and the distance was carefully measured [34–36]. The illumination conditions were verified and systematically checked using the X-Rite i-ONE precision photometer.

The PR test only measures ~0.9–1 cpd at the recommended distance of 1m, and the examination must be conducted at different distances when more cycles per degree need to be examined. The PR chart was performed at 1.67 m rather than the 1.0 m standard distance to achieve a spatial frequency of approximately 1.5 cpd to be comparable to the K-CS test.

We employed the following relation between the viewing distances of the PR optotypes and the respective cpd values:

distance=size_lettertan(cycles_lettercpd)

where: *size_letter* is the size of the optotype (e.g. 4.85cm for the standard PR chart), *cycles_letter* is the number of cycles of the optotype (e.g. ~2.5cycles for the standard PR chart), and *cpd* is the number of cycles per degree of subtended angle at a distance equal to *distance*.

*Pelli-Robson CS test*. When evaluated with the PR test, participants pronounced the letters across and down the chart, starting from the top left corner of the chart through the right side. The examination was performed at 1.67m and then at 3.34 m or vice versa to evaluate contrast sensitivity at 1.5 cpd and 3.0 cpd, respectively. Individual differences in contrast sensitivity has been previously reported so all test distances were kept stable using a tape measure, and once chosen, they were maintained throughout the testing, allowing slight head movements [37–39]. The final triplet at which the patient reads at least two of three letters correctly determines the log CS. Responses of “O” for “C” and conversely were accepted and counted as correct. The forced choice technique was used.

**Fig 1:**
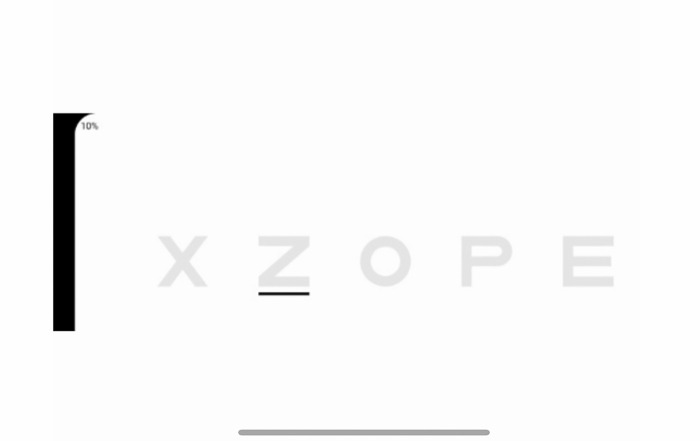
K-CS test.

*K-CS test*. Regarding the K-CS test, the device was switched on approximately 5 min before each examination to allow its output to stabilize. The smartphone was tilted perpendicularly in the line of sight at a 40 cm distance. The examinees held the smartphone-based CS application in landscape mode. Targets of five letters with each specific contrast level were presented on the device luminance (Fig 1). Each spatial frequency was examined separately, starting from 1.5 cpd and proceeding to the 3 cpd with the appropriate letter size. Participants were requested to read loudly the underlined letter. An auditory tone along with a feeling of vibration was produced when the device moves to the next letter. The application detected answers using a certified speech recognizer through an internet connection. The test was set to be terminated once the individual fails to recognize three out of five letters in a row. At the end of the examination, the total score of contrast threshold for each spatial frequency was displayed in log units on the screen.

For both tests, the forced choice technique was performed as well. Subjects were instructed to observe carefully and encouraged to guess using the optimal spectacles [40]. In order to achieve intra-session reliability, all normally sighted and 56 visually impaired participants have been tested twice for both tests within the same session.

### Summary of the characteristics and the examination procedure of the K-CS test

1. The K-CS test presents a single row of five black frequently used Sloan letters in the current literature on a logarithmic scale (ex. H, K, N, O, Z, etc.).
2. The range of contrast is between ~0.1 and 2.06 logarithms of CS.
3. K-CS examines two spatial frequencies of 1.5 and 3.0 cycles per degree corresponding to VA of 1.3 and 1.0 logMAR.
4. The interstimulus drops in contrast sensitivity levels in each step are 0.3 log units.
5. Participants were requested to read loudly the underlined letter. An auditory tone along with vibration was triggered the time the participant vocalized each letter counting the correct answers.
6. The application uses a certified speech recognizer utilizing an internet connection. The test was set to be terminated once the individual makes three out of five letters wrong in a row.
7. The results can be saved and tela-transmitted to the attending physician who has been previously authorized.

### Statistical analysis

The Shapiro-Wilks test was used for the normality assessment. Continuous variables were described using mean (SD) or median (IQR). Categorical variables were described using frequencies (percentages/relative frequencies). Pearson correlation coefficient was used to investigate the relationship between the K-CS test at 3.0 cpd and 1.5cpd as well as between Pelli-Robson for visually impaired patients.

The methods of Bland-Altman were used to investigate the test-retest reliability and the agreement between test versions. The means of the differences and the 95% limits of agreement (LoA) were determined. Spearman’s correlation coefficient was used to examine the associations between the contrast tests administered. Data analysis was conducted using IBM SPSS 27.0 (IBM Corp., Armonk, NY) and R ver.4.0.0 (R Foundation of Statistical Computing, Vienna, Austria).

## Results

### The sample

Group 1 consisted of 46 individuals with age-related macular degeneration, 10 with diabetic retinopathy, 6 with glaucoma, and 5 with retinitis pigmentosa (Table 1). The mean BCVA was 0.67 logMAR (SD = 0.21) and the mean age was 72.72 years (SD = 13.49), including 31 females and 36 males (Table 1). The control group included 50 age-matched normal subjects with mean BCVA 0.0 logMAR and mean age of 71.86 years (SD = 13.49), including 21 females and 29 males (Table 2).

**Table 1:** Distribution of ocular diseases among visually impaired participants.

**Age—related Macular Degeneration, N (%)**	46 (39.3%)
**Glaucoma, N (%)**	6 (5.1%)
**Diabetic Retinopathy, N (%)**	10 (8.5%)
**Retinitis Pigmentosa, N (%)**	5 (4.3%)

N = 67 visually impaired individuals

**Table 2:** Participant characteristics.

	Visually Impaired N = 67 (56.3%)	Control N = 50 (42.7)
**Females, N (%)**	31 (46.3)	21 (42.0)
**Males, N (%)**	36 (53.7)	29 (58.0)

56 visually impaired participants and 50 controls were retested with both tests at 1.5 and 3.0 cpd. They were re-examined the same day [41] and the results are shown in Table 3. Both K-CS and Pelli-Robson test indicate excellent intra-session repeatability (Table 4).

**Table 3:** Mean (±SD) scores of the K-CS test at 1.5 cpd and 3 cpd, in visually impaired and normally sighted participants.

K-CS test	cpd	Test	Retest		
		**Score, mean (SD)**	**Score, mean (SD)**	**Mean Difference** **Test—Retest (SD)**	**95% Limits of Agreement (LoA)**
**Visually Impaired**	1.5 cpd	1.193 (0.289)	1.193 (0.299)	-1.98 x 10^−18^ (0.057)	±0.112
**Normal**	1.5 cpd	1.888 (0.059)	1.888 (0.059)	0	0
**Visually Impaired**	3 cpd	1.00 (0.343)	1.016 (0.354)	-0.016 (0.068)	±0.133
**Normal**	3 cpd	1.828 (0.129)	1.828 (0.129)	0	0

**Table 4:** Mean (±SD) scores of the Pelli-Robson test at 1.5 cpd and 3 cpd, in visually impaired and normally sighted subjects.

PR test	cpd	Test	Retest		
		**Score, mean (SD)**	**Score, mean (SD)**	**Mean Difference** **Test—Retest (SD)**	**95% Limits of Agreement (LoA)**
**Visually Impaired**	1.5 cpd	1.152 (0.314)	1.154 (0.314)	-0.002 (0.073)	±0.143
**Normal**	1.5 cpd	1.932 (0.492)	1.932 (0.492)	0	0
**Visually Impaired**	3 cpd	0.924 (0.350)	0.940 (0.350)	-0.016 (0.093)	±0.183
**Normal**	3 cpd	1.860 (0.742)	1.860 (0.742)	0	0

**Fig 2:**
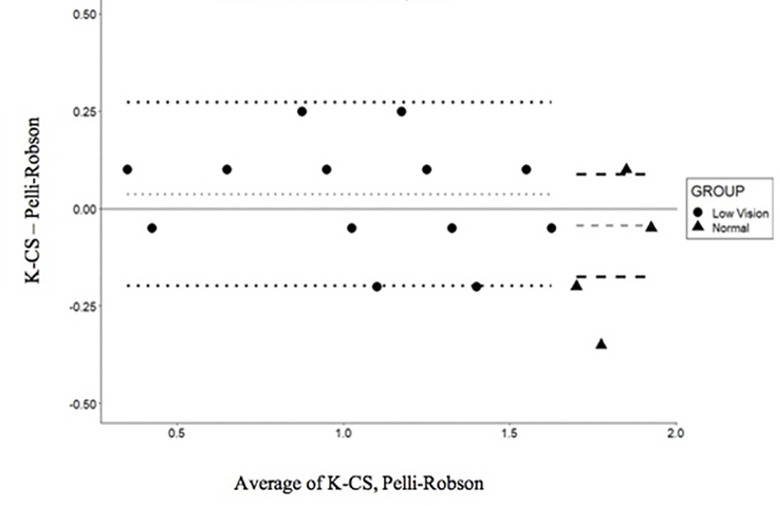
Bland-Altman plot for the differences between the K-CS and PR scores for 1.5 cpd. The dotted and dashed lines show the mean difference (gray) and the 95% LoA (black) for the low-vision and normally sighted subjects, respectively.

**Fig 3:**
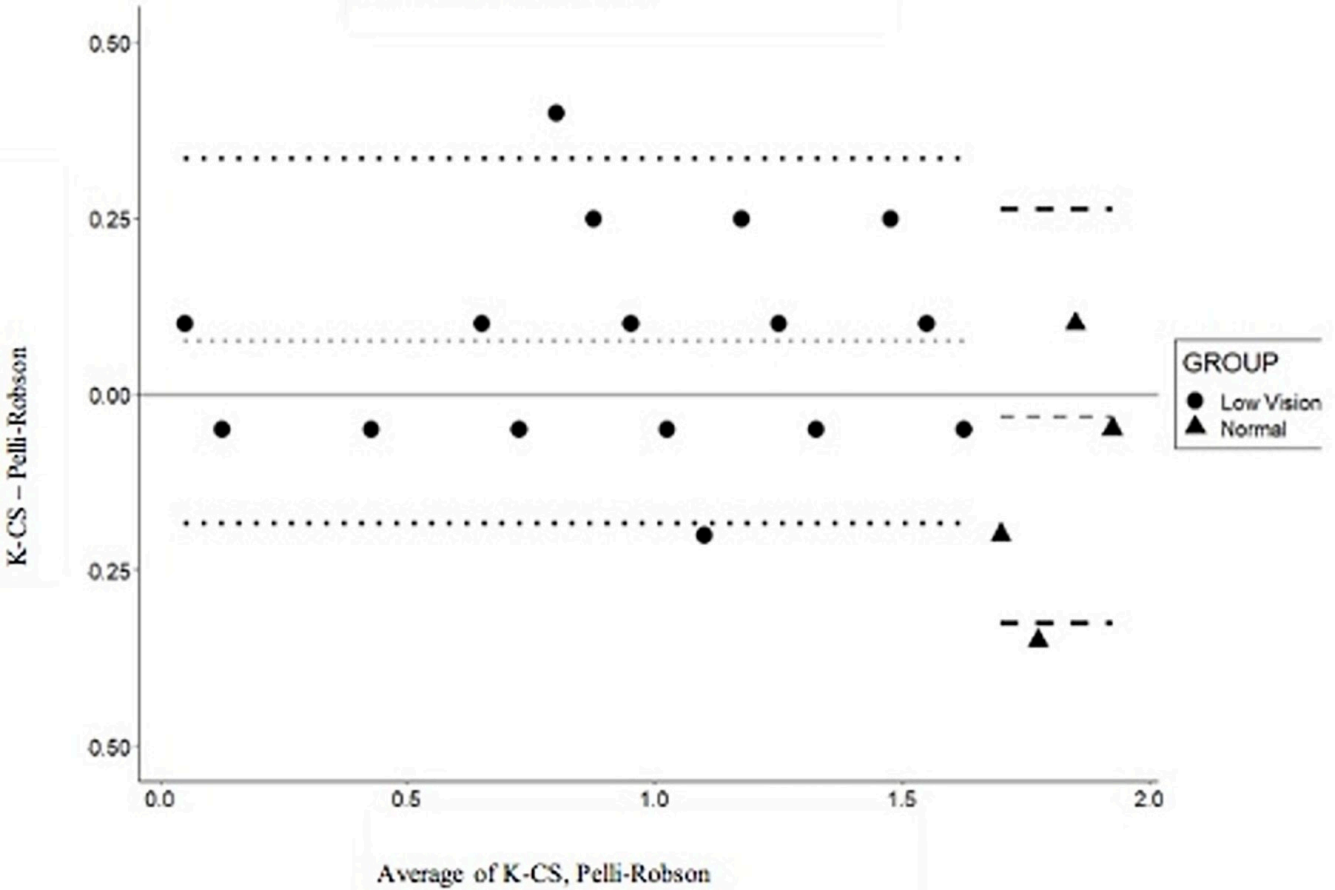
Bland-Altman plot for the differences between the K-CS and PR scores for 3 cpd. The dotted and dashed lines show the mean difference (gray) and the 95% LoA (black) for the low-vision and normally sighted subjects, respectively.

The repeatability of the K-CS and the Pelli-Robson tests in the visually impaired subjects and controls at 1.5 cpd and 3.0 cpd is also shown in Figs 2 and 3, where the test-retest difference is plotted as a function of the mean of the two administrations. The dotted and dashed lines show the mean difference (gray) and the 95% LoA (black) at 1.5cpd for the visually impaired and normally sighted individuals, respectively (Fig 2). The values at 3 cpd are shown in Fig 3.

Mean (SD) contrast sensitivity for both tests in both cycles per degree in visually impaired and control groups is shown in Table 5. The visually impaired group compared to the controls provided worse contrast sensitivity scores in both cycles per degree. In specific, at 1.5 cpd the visually impaired participants, examined through the K-CS and the Pelli-Robson test, had a mean log CS score of 1.19 and 1.15, while controls had a score of 1.908 and 1.93, respectively. At 3cpd, visually impaired individuals had a score of 1.01 and 0.94 through K-CS and Pelli-Robson, while controls had scores of 1.83 and 1.86, respectively as shown in Table 5.

**Table 5:** Mean (±SD) scores of K-CS and Pelli-Robson tests at 1.5 cpd and 3 cpd in visually impaired and normally sighted subjects.

	Cycles per degree (cpd)	K-CS test Score, mean (SD)	Pelli-Robson Score, mean (SD)	Mean Difference K-CS-PR (SD)	95% Limits of Agreement (LoA)
**Visually Impaired**	1.5 cpd	1.19 (0.27) CI:95% 0.66, 1.72	(0.31) CI:95% 0.54, 1.76	0.037 (0.120) CI:95% -0.208, 0.282	±0.235
**Normal**	1.5 cpd	1.908 (0.06) CI:95% 1.79, 2.03	1.93 (0.05) CI:95% 1.83, 2.03	-0.044 (0.067) CI:95% -0.093, 0.181	±0.132
**Visually Impaired**	3 cpd	1.01 (0.33) CI:95% 0.36, 1.66	0.94 (0.34) CI:95% 0.27, 1.61	0.075 (0.132) CI:95% -0.194, 0.344	±0.259
**Normal**	3 cpd	1.83 (0.13) CI:95% 1.58, 2.08	1.86 (0.07) CI:95% 1.72, 1.99	-0.032 (0.150) CI:95% -0.338, 0.274	±0.295

Pearson correlation coefficient was used to investigate the correlation between the K-CS test at 3.0 cpd and 1.5cpd as well as between the Pelli-Robson for visually impaired patients at the same frequencies. There is a significant positive correlation between the K-CS test at 1.5cpd and at 3.0 cpd for visually impaired patients (Pearson r = 0.853, p<0.001). There is also a significant positive correlation between Pelli-Robson at 1.5 cpd and 3.0 cpd for visually impaired patients (Pearson r = 0.880, p<0.001).

## Discussion

This study was designed to evaluate several aspects of CS data in a group of individuals with visual impairment, as well as in a control group. Specifically, our study focused on the performance of the PRCS and the K-CS test, the comparison between them and their intra-session repeatability and reliability. Visually impaired participants exhibited significantly lower CS scores compared to controls for both K-CS and Pelli-Robson tests. Furthermore, both K-CS and Pelli-Robson tests were found to have significant intra-session test-retest reliability under both cycles per degree [41]. The aim of the novel smartphone K-CS test was to serve as a quick and easily accessible method for the evaluation of CS function.

Previous studies of the PR test support that mean values in normally sighted subjects was 1.79 logCS, whereas in the visually impaired was 0.98 for AMD patients and 1.64 for glaucoma patients [42]. Others have reported mean normal values of 1.66 logCS for normal and 1.29 logCS for low vision patients [43]. The performance of the PR test in our study is highly comparable to that reported by previous studies for normally sighted and visually impaired individuals. The K-CS test performed well at 1.5 cpd both for the visually impaired and the controls, with a mean contrast sensitivity score of 1.91 and 1.19 for controls and visually impaired participants, respectively. K-CS mean contrast at 3 cpd in normally sighted individuals was 1.83 and in visually impaired participants 1.01.

CS function is also measured with gratings. Using gratings, sensitivity is evaluated through spatial frequencies that may extend the visible range. Over the few past years, in clinical settings, the best approach to CS function examination is considered the use of appropriate optotypes, though, these letter optotypes may be less accurate [44]. Nevertheless, the letter CS testing is simple for the clinician, and has good repeatability making it suitable for use in clinical studies [45].

Although charts like Pelli-Robson could be reliable tools for assessing contrast sensitivity function; they are infrequently applied in routine clinical practice. They are large and need careful handling as well as require training, equipment, and time. Like all charts, Pelli-Robson has technical issues, including uneven lighting, faded print, and reflective surfaces [41]. Certain mobile electronic devices (e.g. iPad test, Mobile app Aston, PsyPad test etc.) have been developed to assess visual function including the CS function evaluation [46–48]. Nonetheless, the majority of them allow subjects to assess CS via the iOS operating system and were validated mainly through Apple devices and especially on iPad devices. Given the high cost of such devices, these applications are inaccessible to several people. The smartphone-based K-CS test can be administered using an Android device and has features suitable for use. Today, many people have smartphones, which make digital CS assessments much more accessible. In addition, the K-CS takes approximately 1.5 to 2 minutes per cpd, for each eye, making it faster than many existing digital or printable contrast sensitivity tests. However, for research purposes various tests are comparably used [49].

Towards developing a quick method for self-assessing the CS function for visually impaired individuals, the steps of 0.30 log units of contrast that we applied on the K-CS test were based on previous reports that changes of 0.25 log units are clinically meaningful [43]. By assessing visually impaired individuals and to be comparable with the gold standard Pelli-Robson, we chose to examine participants at 1.5 cpd. Participants were also examined at 3 cpd since it is considered to be the region of peak sensitivity. The intra-session repeatability of both tests was high, and the K-CS scores highly correlated with those of the PR CS test at both 1.5 and 3 cycles per degree. There are various research attempts to exploit the clinical information provided by the CS function examination [50–53]. Given the fact that in some cases the correlation of VA and CS is not strong, the incorporation of a quick and reliable CS function test such as the K-CS test in clinical practice could potentially address the limitations of high contrast VA examination [20, 54].

As a novel smartphone-based test, the K-CS has advantages along with a few limitations. Even though the step of 0.30 log CS is of clinical significance when assessing individuals with visual impairment, it is not as precise as a step of 0.15 logCS units, utilized by the PR chart. The new test aims to serve as a simple and quick test for CS function evaluation.

Overall, the results suggest that the K-CS test is appropriate for an easy and quick self-performed test of CS function in visually impaired and normally sighted populations in clinical settings. The self-performance in the clinical setting provided comparable results to the PR test and may serve as an additional tool for ophthalmologists. Further studies to assess the performance requirements at home settings are in progress.

## Conclusions

The K-CS provides the advantages of reduced testing time along with reliable results, therefore serving as an alternative tool for evaluating the CS function in consecutive examinations with the cooperation of the physician in clinical settings. Further investigation is in progress concerning the estimation of the prerequisites of performance in home settings.

## Supporting information

S1 Table Discrete contrast levels (log-units) produced by the Samsung A30S mobile device. (DOCX)

S1 Data (XLSX)
