# Supplementary material for: Evaluation of contrast sensitivity in visually impaired individuals using K-CS test. A novel smartphone-based contrast sensitivity test–Design and validation
Source: PLoS One. 2024 Feb 8;19(2):e0288512. doi: 10.1371/journal.pone.0288512 (PMC10852338; doi:10.1371/journal.pone.0288512)
Supplement: S1 Table — (DOCX) [file pone.0288512.s001.docx]

**S1 Table . Discrete Contrast levels (log-units) produced by the Samsung A30S mobile device**

| LEVEL | Contrast level (log-units) |
| --- | --- |
| 1 | 2.06 |
| 2 | 1.76 |
| 3 | 1.51 |
| 4 | 1.4 |
| 5 | 1.34 |
| 6 | 1.26 |
| 7 | 1.2 |
| 8 | 1.15 |
| 9 | 1.09 |
| 10 | 1.04 |
| 11 | 1 |
| 12 | 0.97 |
| 13 | 0.94 |
| 14 | 0.91 |
| 15 | 0.88 |
| 16 | 0.85 |
| 17 | 0.83 |
| 18 | 0.81 |
| 19 | 0.79 |
| 20 | 0.77 |
| 21 | 0.75 |
| 22 | 0.73 |
| 23 | 0.71 |
| 24 | 0.69 |
| 25 | 0.67 |
| 26 | 0.66 |
| 27 | 0.65 |
| 28 | 0.63 |
| 29 | 0.62 |
| 30 | 0.61 |
| 31 | 0.59 |
| 32 | 0.58 |
| 33 | 0.57 |
| 34 | 0.56 |
| 35 | 0.55 |
| 36 | 0.53 |
| 37 | 0.52 |
| 38 | 0.51 |
| 39 | 0.5 |
| 40 | 0.49 |
| 41 | 0.48 |
| 42 | 0.47 |
| 43 | 0.46 |
| 44 | 0.45 |
| 45 | 0.44 |
| 46 | 0.43 |
| 47 | 0.42 |
| 48 | 0.41 |
| 49 | 0.4 |
| 50 | 0.39 |
| 51 | 0.38 |
| 52 | 0.37 |
| 53 | 0.36 |
| 54 | 0.35 |
| 55 | 0.34 |
| 56 | 0.33 |
| 57 | 0.32 |
| 58 | 0.31 |
| 59 | 0.3 |
| 60 | 0.29 |
| 61 | 0.28 |
| 62 | 0.27 |
| 63 | 0.26 |
| 64 | 0.25 |
| 65 | 0.24 |
| 66 | 0.23 |
| 67 | 0.22 |
| 68 | 0.21 |
| 69 | 0.2 |
| 70 | 0.19 |
| 71 | 0.18 |
| 72 | 0.17 |
| 73 | 0.16 |
| 74 | 0.15 |
| 75 | 0.14 |
| 76 | 0.13 |
| 77 | 0.12 |
| 78 | 0.11 |
| 79 | 0.1 |
| 80 | 0.09 |
| 81 | 0.08 |
| 82 | 0.07 |
| 83 | 0.06 |
| 84 | 0.05 |
| 85 | 0.04 |
| 86 | 0.03 |
| 87 | 0.02 |
| 88 | 0.01 |
| 89 | 0 |
